# Economic costs of hospitalized diarrheal disease in Bangladesh: a societal perspective

**DOI:** 10.1186/s41256-017-0056-5

**Published:** 2018-01-05

**Authors:** Abdur Razzaque Sarker, Marufa Sultana, Rashidul Alam Mahumud, Nausad Ali, Tanvir M Huda, M. Salim uzzaman, Sabbir Haider, Hafizur Rahman, Ziaul Islam, Jahangir A. M. Khan, Robert Van Der Meer, Alec Morton

**Affiliations:** 1 International Centre for Diarrhoeal Disease Research, Bangladesh (icddr,b), Dhaka, Bangladesh; 2 University of Strathclyde, Glasgow, UK; 3 Sydney School of Public Health, Sydney Medical School, The University of Sydney, Sydney, Australia; 4 Institute of Epidemiology, Disease Control and Research, Dhaka, Bangladesh; 5 Health Economics Unit, Ministry of Health and Family Welfare, Dhaka, Bangladesh; 6 Liverpool School of Tropical Medicine, Liverpool, UK

**Keywords:** Bangladesh, Catastrophic expenditure, Cost-of-illness, Diarrhea, Out-of-pocket payment, Public hospitals

## Abstract

**Background:**

Diarrheal diseases are a major threat to human health and still represent a leading cause of morbidity and mortality worldwide. Although the burden of the diarrheal diseases is much lower in developed countries, it is a significant public health problem in low and middle-income countries like Bangladesh. Though diarrhea is preventable and managed with low-cost interventions, it is still the leading cause of morbidity according to the patient who sought care from public hospitals in Bangladesh indicating that significant resources are consumed in treating those patients. The aim of the study is to capture the inpatients and outpatient treatment cost of diarrheal disease and to measure the cost burden and coping mechanisms associated with diarrheal illness.

**Methods:**

This study was conducted in six randomly selected district hospitals from six divisions (larger administrative units) in Bangladesh. The study was performed from the societal perspective which means all types of costs were identified, measured and valued no matter who incurred them. Cost analysis was estimated using the guideline proposed by the World Health Organization for estimating the economic burden of diarrheal diseases. The study adopted quantitative techniques to collect the household and hospital level data including structured and semi-structured questionnaires, observation checklists, analysis of hospital database, telephone interviews and compilation of service statistics.

**Results:**

The average total societal cost of illness per episode was BDT 5274.02 (US $ 67.18) whereas the average inpatient and outpatient costs were BDT 8675.09 (US $ 110.51) and BDT 1853.96 (US $ 23.62) respectively. The cost burden was significantly highest for poorest households, 21.45% of household income, compared to 4.21% of the richest quintile.

**Conclusions:**

Diarrheal diseases continue to be an overwhelming problem in Bangladesh. The economic impact of any public health interventions (either preventive or promotive) that can reduce the prevalence of diarrheal diseases can be estimated from the data generated from this study.

## Background

Diarrheal diseases are a global public health problem and a leading cause of morbidity and mortality across the world. According to the latest Global Burden of Disease Study, about 2.39 billion of diarrheal cases occurred globally and approximately 0.53 million of under five children died every year [1, 2]. Specifically, incidence and case-fatality ratios are much higher in lower and middle income (LMI) countries [3]. In Bangladesh, diarrhea diseases are still very common among children under 5 years old [4]. In developing countries, diarrhea-related morbidity and mortality is directly linked with limited access to potable water and proper sanitation system [5]. Several studies observed that epidemics of diarrheal disease are associated with episodes of flooding [6], socioeconomic status [7], urban status [8] high population density, low education level and the proximity of household clusters to contaminated surface water [9–11]. The diseases are highly sensitive to climate, showing seasonal variations in numerous sites [12]. Relative humidity and temperature influence the rate of replication of different types of pathogens such as bacteria and protozoa, and also the survival of enteroviruses in the environment which is another cause of diarrheal diseases [13]. Diarrhea is an alteration in normal bowel movement characterized by an increase in water content, volume, or frequency of stools [14]. If the disease lasts “more than 7 days” and “at least 14 days” the term “prolonged” and “persistent” diarrhea is used respectively [15, 16].

Though diarrhea is preventable and managed with low-cost interventions, it is still the top cause of morbidity for patients who sought care from the public hospital system in Bangladesh [17] and significant resources are expended in treating these patients. Diarrheal diseases affect people of all ages irrespective of their socio-economic status and are particularly prevalent among poor people. A significant cause for concern in Bangladesh is that approximately 26% of the people are below the poverty datum line. In some cases, the episode can be managed at home and does not require hospital treatment. However, considering the direct and indirect cost of households, it represents a substantial economic burden for the affected households [18].

There are several studies about the economic burden of diarrheal disease in many countries [19–25] but knowledge about the treatment cost of a full diarrheal episode is still limited in Bangladesh although such studies are vital for informing policies and allowing international comparisons [26, 27]. There are several economic studies are available focusing diarrheal diseases in Bangladesh [28–31]. Nevertheless, these studies did not consider the societal perspective to capture the average cost for diarrheal treatment. However, it is essential for policy makers to understand the precise estimate of the economic cost of diarrheal treatment based on uniform methodologies for setting priorities for health sector as well as for balanced allocation of scarce resources. The intent of this study is to estimate the age and sex specific economic costs of diarrheal disease considering a broad social perspective and to capture the healthcare seeking pattern during the diarrheal episode in Bangladesh.

## Methods

### Study setting and sample

This study was conducted in public district hospitals in Bangladesh. Public hospitals play a major role in providing treatment for a relatively large population as the treatment cost in public hospitals is less than private for-profit hospitals and hospitals financed by non-governmental organisations (NGOs). A total of 801 diarrheal patients were randomly selected and interviewed from January to December 2015.

### Study perspective

The study was a societal perspective which means all types of costs were identified, measured and valued no matter who incurred them. The societal perspective is the summation of provider and household perspective which is recommended in the current standards for cost-effectiveness analysis methods [32, 33].

### Cost estimates

Cost analysis was estimated using the guideline proposed by the World Health Organization (WHO) for estimating the economic burden of diarrheal diseases [34]. A bottom-up micro-costing approach was used to generate the cost of illness per episode per patient where all relevant cost components are identified and valued at the most detailed level [34, 35].

To capture the household economic cost of illness both direct and indirect costs were captured. *Direct costs* were defined as expenditure during treatment by households which consists of two parts: direct medical cost and direct non-medical cost. Direct medical expenses include those costs consumed for healthcare resources during diarrheal episodes such as medicine, diagnosis, registration fees and others. The direct non-medical cost includes transportation, lodging, food items, informal payment, payment for helping the patients during treatment. There are other types of expenditure such as material costs like a mug, jar, plate, glass and other items such as a coil, lighter and other cost items of patients, as well as expenditure for people accompanying the patients and their caregivers to stay outside of hospitals during diarrheal treatment.

The *indirect cost* was considered the income loss as well as productivity loss because of travel to the health centre and costs due to absence from work because of illness related to the diarrheal disease. Self-reported wage rates were used for estimating the income loss. Productivity costs were estimated using a human capital approach which reflected the value of all unpaid time devoted to caregiving themselves, as well as family members and friends [36]. The inclusion of caregiving time based on the assumption that time dedicated to caregiving may represent foregone non-market activities such as school, household chores, child care, and leisure or domestic work [37, 38]. This time comprised time spent directly on patient care (by the patient and by unpaid attendants or caregivers), such as attending to diarrhea-related health care appointments. To capture the productivity losses for non-market activities, we used the age-specific and occupation-specific wage rates [38, 39]. We used age-specific wages for adults, teenagers and children aged 5 to 14 years, where the minimum salary rate according to national level was given by the adult patients, one-half for the teenagers and three-quarters to capture productivity loss for children, however, half of the average salary rate assigned to unpaid home workers considering their age group [38, 39]. Intangible or psychic costs such as costs related to suffering and grief were not measured in this study as those costs are not valued in the disease-specific cost of illness research [38, 40]. Again, time cost of visitor and extra irregular expense borne by the patients, caregivers, and visitors on their way during the time of hospitalization were not included in the analysis. The household cost burden measured by the percentage of total household earnings that was consumed by the treatment cost of diarrheal diseases [41].

The average treatment costs for diarrheal diseases borne by the public hospitals were measured using the patient-specific treatment costs approach according to WHO guidelines [34]. In this aspect, average outpatients and inpatients visits costs were estimated. The costs included costs of diagnosis, laboratory cost, medicine costs, feeding costs, institutional cost and other associated costs borne by the hospitals for treating on a patient-specific basis. Shared costs were allocated according to the number of patient’s days of hospitalization. Capital cost was annuitized with 3% discount rate [35]. The provider actual cost of illness calculated the provider’s cost for treatment devoid of any fees received from the patients for hospitalization, drug, diagnostic tests, etc. Finally, the societal cost of illness was estimated by adding provider’s actual cost of illness per patients with the cost incurred per household.

### Method of data collection

The study adopted quantitative techniques to collect the household and hospital level data including the structured questionnaire, observation checklist, hospital database, telephone interviews and compilation of service statistics. For household level, respondents were the adult patients or the accompanying person who was most familiar with the costs incurred during the treatment of the patient and interviews were conducted during discharge from the hospital. Patients’ records drawn from the above Hospital Records Departments (HRD) were reviewed for the use of resources for diarrheal patients. Resource utilisation data were abstracted from the registers for inpatients or outpatients. At the central level, several offices such as finance, procurement and supply and maintenance unit were contacted to validate the cost information. A research assistant reviewed the patient’s records, and data abstraction forms were updated daily until the discharge of the patient. Finally, a telephone interview was conducted for taking necessary information within 1 week after discharge from hospital. Caregivers were also interviewed in their language of preference with the use of a standardised interview schedule on admission or soon thereafter. Questions were asked regarding transportation, consultation before the hospital visit, medicine brought, expenses during the hospitalization, and losses of wages resulting from absence from work.

### Ethics approval

The research protocol of this study was approved by the Institutional Review Board of the International Centre for Diarrheal Disease Research, Bangladesh (icddr,b). Informed consent was obtained from all respondents before data collection.

### Data analysis

Completed questionnaires were developed by a qualified supervisor with both numerical and logical checks to minimise errors. Before analysis, missing answers and outliers were systematically verified. Patient specific cost of illness borne by the household and provider costs are reported separately. The data were analysed using a spreadsheet in Microsoft Excel and Stata/SE 13.0 (StataCorp. College Station, TX, USA). Proportion, frequencies, rates and ratio, were presented with a standard deviation in local currency, i.e., Bangladeshi Taka (BDT) and US dollars (US$) applying the exchange rate (US$1 = 78.5 BDT) during the year of the survey mid 2014- mid 2015 [42]. Like the earlier study, to test the robustness of the assumption, a sensitivity analysis was conducted to examine the impact of potential outlier on the total cost of illness [43]. However, the cost of caregivers of households had a higher level of uncertainty [44]. For this purpose, we tested the effect of changes of 20% in the parameter values of both direct and indirect cost of households and 20% change of both medical and non-medical cost of the provider as performed in other studies [38, 45].

## Results

### Background characteristics

A total of 801 patients participated in the study from selected public district hospitals, among whom 402 and 399 patients were inpatients and outpatients respectively. All respondents participating in this survey were provided with information about the study, and none withheld consent. The average age of the patients were 15.46 years (SD = 21.08 years) of which 57.43% were age under 5 years followed by 24.34% for age ranged 15-45 years. The highest percentage of patients were homemakers (38.12%), students (23.75%), self-employed (14.37%) and only 9.09% of the patients were salaried employees. Approximately, 31% of the patients had up to secondary grade education, and primary school (29.64%). Only 4.19% of the patients had higher level education whereas 18.56% had no formal education, and 11.08% had no education (Table 1). However, diarrheal occurrence was higher among households with lower parental levels of educational attainment (Table 1). It was also higher among households with up to 4 to 5 members (43.32%) followed by more than five members (38.83%), and the average patient’s household size was 3.20 (SD = 0.74) (Table 1). The average monthly income and expenditure of the household were BDT 19,603 (US$ 249.72) and BDT 15,470 (US$ 197.07) respectively while the average household healthcare expenditure in the previous 3 months was BDT 5191 (US$ 66.13) (Table 1).

**Table 1:** Background characteristics of the study participants for public tertiary level hospital (*N* = 801)

Variables	Description	n (%) / mean ± SD	95% CI (% or mean)
Number of patients	N	801	
	Inpatient	402 (50.19)	(46.72, 53.65)
	Outpatient	399 (49.81)	(46.35, 53.28)
Patient age (%)	Up to 4	460 (57.43)	(53.09, 59.96)
	5 to 14	55 (6.87)	(6.08, 9.81)
	15 to 45	195 (24.34)	(21.49, 27.44)
	46 to 60	62 (7.74)	(6.08, 9.81)
	60+	29 (3.62)	(2.53, 5.17)
Patient age yrs. (mean ± SD)	Up to 4	1.41 ± 0.96	(1.32, 1.50)
	5 to 14	8.12 ± 2.76	(7.38, 8.87)
	15 to 45	29.08 ± 9.43	(27.74, 30.41)
	46 to 60	55.47 ± 4.3	(54.38, 56.56)
	60+	75.03 ± 11.99	(70.47, 79.6)
	Overall	15.46 ± 21.08	(13.99, 16.92)
Gender (%)	Male	404 (50.44)	(46.97, 53.9)
	Female	397 (49.56)	(46.1, 53.03)
Patient Occupation (%)	House wife	130 (38.12)	(33.09, 43.42)
	Students	81 (23.75)	(19.51, 28.59)
	Self-employment	49 (14.37)	(11.02, 18.53)
	Unemployed	7 (2.05)	(0.98, 4.26)
	Salaried employee	31 (9.09)	(6.45, 12.66)
	Business	18 (5.28)	(3.34, 8.24)
	Others	25 (7.33)	(4.99, 10.64)
Patient education level (%)	Illiterate	37 (11.08)	(8.17, 15.03)
	No formal education	62 (18.56)	(14.82, 23.26)
	Up to primary	99 (29.64)	(24.55, 34.36)
	Secondary	103 (30.84)	(26.26, 36.23)
	Higher secondary	19 (5.69)	(3.67, 8.82)
	Higher	14 (4.19)	(2.51, 7.01)
Mother education level (%)	Illiterate	156 (19.48)	(16.87, 22.37)
	formal education	114 (14.23)	(11.98, 16.83)
	Up to primary	199 (24.84)	(21.97, 27.96)
	Secondary	274 (34.21)	(30.99, 37.57)
	Higher secondary	37 (4.62)	(3.36, 6.31)
	Higher	21 (2.62)	(1.71, 3.99)
Father education level (%)	Illiterate	139 (17.35)	(14.88, 20.14)
	No formal education	139 (17.35)	(14.88, 20.14)
	Up to primary	191 (23.85)	(21.01, 26.93)
	Secondary	241 (30.09)	(27, 33.36)
	Higher secondary	49 (6.12)	(4.65, 8.01)
	Higher	42 (5.24)	(3.9, 7.02)
Household size (%)	Less than 2	6 (0.75)	(0.34, 1.66)
	2 to 3	137 (17.1)	(14.65, 19.88)
	4 to 5	347 (43.32)	(39.92, 46.79)
	More than 5	311 (38.83)	(35.5, 42.26)
Household size		3.20 ± 0.74	(3.15, 3.25)
Patient monthly income, BDT (*n* = 411)		3976.78 ± 8397.02	(2974.40, 4979.16)
Monthly income of household (BDT)		19,603.37 ± 26,641.74	(17,755.58, 21,451.16)
Monthly expenditure of household (BDT)		15,469.69 ± 10,702	(14,727.43, 16,211.94)
Overall healthcare expenditure last 3 months (BDT)		5191.43 ± 17,745.43	(3960.66, 6422.20)
Income quintile (BDT)			
Poorest quintile (≤10,000 BDT)		7963.77 ± 2025.22	(7723.785, 8203.75)
2nd quintile (10,001- 12,000)		11,920.73 ± 266.08	(11,862.27, 11,979.20)
3rd quintile (12,001-18,000)		15,227.71 ± 1253.44	(15,035.63, 15,419.80)
4th quintile (18,001-30,000)		23,540.11 ± 4014.05	(22,961.02, 24,119.20)
Upper quintile (30,000+)		62,188.89 ± 62,881.18	(49,018.68, 75,359.10)

### Distribution of average household cost of illness

Table 2 shows the average cost of illness per diarrheal episode from the household’s perspective. The average total costs for treating the diarrhea patients were BDT 4178.68 (US $ 53.23). The average total out-of-pocket (OOP) cost was BDT 1688.17 (US $ 21.51) which represented 40% of the total household cost; where 28% was the direct medical and 12.41% was the direct non-medical cost. For OOP costs, medicine was the highest cost driver (BDT 1064.19 or US $13.56) followed by non-medical transportation cost (BDT 246.58 or US$ 3.14). Among the direct medical costs, diagnostic costs (BDT 37.63 or US$ 0.48) and consultation fee (BDT 26.37 or US $0.34) were the two most significant cost driver during the episode. However, caregivers expenditure (BDT 127.87 or US $1.63) was the critical cost component of direct non-medical costs, which included transportation, food, mobile bill and other related expenses borne by the caregivers during the episodes of diarrhea. For the indirect costs per episode (BDT 2490 or US $ 31.72) caregivers income loss was the highest (BDT 2, 179.50 or US$ 27.76), higher than patient’s productivity loss (BDT 310.51 or US$ 3.96).

**Table 2:** Distribution of average household cost of diarrheal treatment for tertiary level hospital (*N* = 801) BDT (US$)

Cost	Parameter	Overall cost of treatment		Proportion of total cost
		Average	SD	
Direct Medical	Diagnostic	37.63 (0.48)	189.71 (2.42)	28
	Medicine	1064.19 (13.56)	1427.04 (18.18)	
	Consultant fee	26.37 (0.34)	121.38 (1.55)	
	Registration/admission fee	14.54 (0.19)	11.42 (0.15)	
	Medical materials (syringe/cannula etc)	27.32 (0.35)	62.43 (0.80)	
	Bed/ Cabin charge	0.28 (0.00)	7.95 (0.10)	
Direct Non-Medical	Transportation cost	246.58 (3.14)	427.76 (5.45)	12.41
	Food items	113.82 (1.45)	239.77 (3.05)	
	Informal payment	7.40 (0.09)	22.65 (0.29)	
	Caregiver’s payment	0.01 (0.00)	0.35 (0.00)	
	Materials (mug/glass etc.)	22.65 (0.29)	70.59 (0.90)	
	Lodging	0.00 (0.00)	0.01 (0.00)	
	Caregivers expenditure	127.87 (1.63)	456.80 (5.820	
Total direct cost		1688.17 (21.51)	2010.95 (25.62)	40.4
In-direct cost	Patient income loss	310.51 (3.96)	1374.40 (17.51)	
	Caregiver’s income loss	2179.50 (27.76)	3445.12 (43.89)	
Total indirect cost		2490.01 (31.72)	3881.48 (49.45)	59.6
Total cost		4178.68 (53.23)	5166.20 (65.81)	100

### Household cost and associated variable

Table 3 shows the association between the cost of illness and the variables of interest. The average household cost of illness was significant among the age groups. The average cost of illness (BDT 8407.58 or US$ 107.1) for an elderly person was comparatively higher than any other age group. However, the cost of treating under 5 year old children were significantly lower (BDT 3440.66 or US $ 43.83) than those aged more than 5 years (BDT 5173.09 or US$ 65.90) (*P < 0.001*). The average cost of illness for male patients (BDT 4441.82 or US$ 56.58) was higher than that of females (BDT 3909.9 or US$ 49.81) and was not statistically significant (*P = 0.505*). Furthermore, the cost of illness for inpatient care was significantly (*P < 0.001*) higher (BDT 6570 or US $ 83.7) than that of outpatient care (BDT 1767.58 or US $ 22.52).

**Table 3:** Association between household cost and other variables

Variables	Number of patients (N)	Household cost, BDT (US $)		t / F-statistic	*P*-value
		Average	SD		
Age group (years)					
Up to 4	460	3440.66 (43.83)	4549.87 (57.96)	14.43^a^	<0.0001
5 to 14	55	2483.17 (31.63)	3113.52 (39.66)		
15 to 45	195	4963.35 (63.23)	4766.65 (60.72)		
46 to 60	62	6706.05 (85.43)	8555.34 (108.99)		
60+	29	8407.58 (107.10)	5987.61 (76.28)		
Age group under five and others					
*Under 5 years old patients*	460	3440.66 (43.83)	4549.87 (57.96)	4.60^b^	<0.0001
*More than 5 years old patients*	341	5173.09 (65.90)	5753.68 (73.30)		
Sex					
*Male*	404	4441.82 (56.58)	5581.65 (71.10)	1.46^b^	0.14
*Female*	397	3909.90 (49.81)	4696.13 (59.82)		
Type of care					
*Inpatient care*	402	6570.79 (83.70)	5457.71 (69.52)	14.88^b^	<0.0001
*Outpatient care*	399	1767.58 (22.52)	3465.92 (44.15)		
Income quintile					
Poorest quintile (≤10,000)	276	3689.3 (47.00)	4412.93 (56.22)	0.28^a^	0.89
2nd quintile (10,001- 12,000)	82	4037.95 (51.44)	4785.6 (60.96)		
3rd quintile (12,001-18,000)	166	4202.38 (53.53)	4866.93 (62.00)		
4th quintile (18,001-30,000)	187	4453.03 (56.73)	5789.56 (73.75)		
Upper quintile (30,000+)	90	5189.48 (66.11)	6549.86 (83.44)		
For under 5 years old patients					
Sex					
*Male*	248	3571.68 (45.50)	4566.33 (58.17)	0.67^b^	0.505
*Female*	212	3287.38 (41.88)	4536.52 (57.79)		
Type of care					
*Inpatient care*	142	6770.96 (86.25)	4376.74 (55.75)	11.36^b^	<0.0001
*Outpatient care*	318	1953.54 (24.89)	3777.22 (48.12)		
Income quintile					
Poorest quintile (≤10,000)	167	3499.31 (44.58)	4596.37 (58.55)	0.28^a^	0.8919
2nd quintile (10,001- 12,000)	48	3835.03 (48.85)	5304.53 (67.57)		
3rd quintile (12,001-18,000)	94	3076.29 (39.19)	3794.47 (48.34)		
4th quintile (18,001-30,000)	111	3554.23 (45.28)	5024.53 (64.01)		
Upper quintile (30,000+)	40	3263.63 (41.57)	3697.33 (47.10)		

^a^One-way analysis of variance (ANOVA) was performed to derive significance level

^b^Independent two samples t-test was performed to derive significance level

### Cost burden and coping strategies

**Fig. 1:**
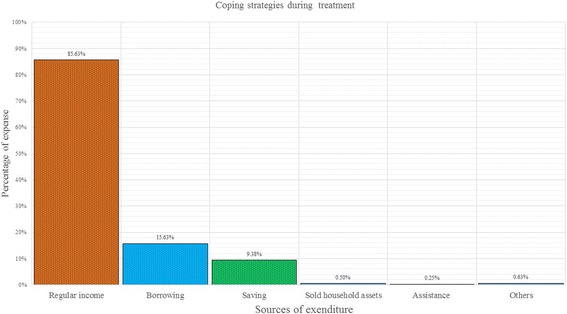
Coping mechanisms during diarrheal treatment

The cost burden of diarrheal illness is presented in Table 4 and the ‘total out of pocket costs’ during treatment is shown as a percentage of the monthly earnings of the households. The OOP payment as a proportion of household income differed significantly among the income groups (*P* < 0.0001). It was observed that during the treatment course, the most common coping strategies were regular income (85.63%) borrowing from others (15.63%) and savings (9.38%) (Fig. 1).

**Table 4:** Cost burden and catastrophic health expenditure in different socioeconomic condition

Income group	Direct cost as percentage of monthly household income	Percentage of household spending for healthcare expenditure as a share of monthly household income			
		10%	15%	20%	25%
Poorest quintile (≤10,000)	21.45% (17.32%-25.58%)	49.20% (43.66%-54.75%)	40.51% (35.18% - 46.08%)	31.83% (26.88% - 37.23%)	27.01% (22.35% - 32.23%)
2nd quintile (10,001- 12,000)	11.6% (9.18%-14.02%)	39.64% (30.94% - 49.05%)	26.13% (18.77% - 35.12%)	16.22% (10.43% - 24.33%)	9.91% (5.55% - 17.06%)
3rd quintile (12,001-18,000)	9.35% (7.92%-10.79%)	31.84% (26.04%-38.26%)	16.14% (11.86%-21.59%)	10.31% (6.94% - 15.06%)	7.17% (4.43% - 11.41%)
4th quintile (18,001-30,000)	6.45% (5.27%-7.64%)	20.62% (16.10%-26.02%)	10.12% (6.97% - 14.46%)	5.84% (3.54% - 9.47%)	2.33% (1.05% - 5.11%)
Upper quintile (30,000+)	4.21% (3.34%-5.08%)	8.22% (4.71%-13.95%)	4.79% (2.29% - 9.75%)	4.11% (1.85% - 8.87%)	1.37% (0.34% - 5.34%)
Overall	11.75% (10.37%-13.14%)	31.77% (29.02% - 34.66%)	21.37% (18.99% - 23.97%)	15.36% (13.30% - 17.68%)	11.35% (9.57% - 13.43%)
Rich–poor ratio	0.196	0.167	0.118	0.129	0.051
Rich–poor difference	−17.240	−40.980	−35.720	−27.720	−25.640

The overall OOP expenditure due to diarrheal treatment was 11.75% of monthly household income. However, in the poorest quintile, it exceeded 17% of the total household income. The richest (5th) quintile only spent 4.21% of their household income. Considering a 10% threshold level, approximately 32% households suffered from catastrophic expenditure while the poorest quintile suffered more (49%). Even at the highest threshold level of 25%, the poorest 27% of households suffered from catastrophic expenditure due to diarrheal diseases (Table 4).

### Waiting and travel time

**Fig. 2:**
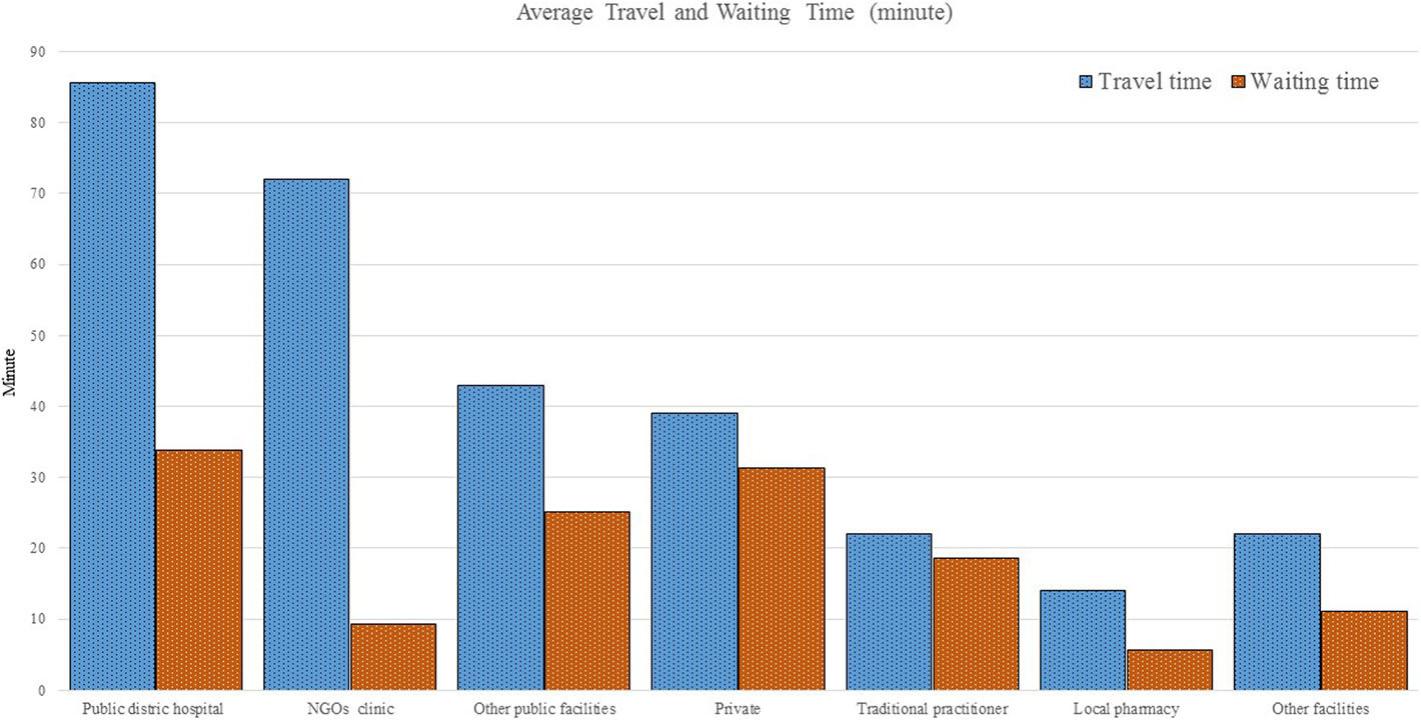
Average travel and waiting time for receiving care

Before coming to the selected public hospitals, most patients received treatment from other formal and informal care providers (Fig. 2). However, in LMI countries like Bangladesh, diarrheal patients are often inadequately treated at home. Homecare is associated with poor outcomes and timely medical treatment is the precondition to minimise the length of each episode and reduce mortality [46]. The average travel time to the public hospital was nearly 2 h and at least 30 min waiting time before being attended to.

### Inpatient and outpatient cost: Hospital perspective

Table 5 shows the average total inpatient and outpatient treatment cost due to diarrheal disease. The average inpatient treatment cost per patient was BDT 2104.09 or US $ 26.80 whereas direct medical costs constituted only 4.18%. The total direct medical cost per diarrheal episode was BDT 88.27 or US $ 1.12, and medicine cost was the largest (BDT 83.23 or US $ 1.06). Among the direct non-medical costs staff salaries was a major cost driver (BDT 836.38 or US $ 10.65) followed by capital costs (BDT 456.31 or US $ 5.81). The other two larger cost components were food costs (BDT 323.25 or US $ 4.12) and space for providing patient services (BDT 321.74 or 4.10).

**Table 5:** Distribution of provider costs: average inpatient vs outpatient’s cost, BDT (US $)

Cost	Parameters	Inpatient (*N* = 402)		Outpatient (*N* = 399)	
		Amount	% of total cost	Amount	% of total cost
Direct Medical	Medicines	83.23 (1.06)	–	28.16 (0.36)	–
	Diagnosis	–	–	–	–
	Disposable items	5.03 (0.06)	–	–	–
Total Direct Medical		88.27 (1.12)	4.18%	28.16 (0.36)	32.56%
Direct Non-Medical	Staff salaries	836.38 (10.65)	–	25.49 (0.32)	–
	Transport	31.39 (0.4)	–	–	–
	Food	323.25 (4.12)	–	–	–
	Stationery	3.44 (0.04)	–	0.1 (−)	–
	Window shade	1.21 (0.02)	–	0.03 (−)	–
	Electricity	9.96 (0.13)	–	0.27 (−)	–
	Gas bill	0.33 (−)	–	0.01 (−)	–
	Water bill	0.01 (−)	–	–	–
	Telephone bill	0.14 (−)	–	–	–
	Other Misallocations	31.66 (0.4)	–	0.85 (0.01)	–
	Capital items	456.31 (5.81)	–	4.84 (0.06)	–
	Building	321.74 (4.1)	–	26.2 (0.33)	–
Total Direct Non-Medical		2015.82 (25.68)	95.82%	57.8 (0.74)	67.44%
Average cost per Patient		2104.09 (26.80)	100%	85.97 (1.1)	100%

The average total outpatient cost was BDT 85.97 or US $1.10 where medical and non-medical costs constituted 32.56 and 67.44% respectively. Medicine cost (BDT 28.16 or US $ 0.36) was the main cost driver followed by the cost of space (BDT 26.20 or US $ 0.33) and staff salaries (BDT 25.49 or US $ 0.32). A lump sum amount of capital cost (BDT 4.84 or US $ 0.06) was also incurred during the treatment course of outpatients (Table 5).

### Societal cost of illness

The average total societal cost of illness per episode was BDT 5274.02 (US $ 67.18) whereas average inpatient and outpatient costs were BDT 8675.09 (US $ 110.51) and BDT 1853.96 (US $ 23.62) respectively (Table 6). Among all of the cost segments, households cost contributed a larger portion (80% of the total costs) and OOP contributed 32% of the total societal cost of illness. Considering the provider actual treatment cost, the non-medical cost (19.66%) was the main cost driver. However, among all of the cost components, the indirect cost of patients and caregivers (BDT 2490 or US $ 31.72) was the main cost driver of all types of care which is not apparent if only provider costs are measured.

**Table 6:** Societal cost of illness due to diarrheal disease, BDT (US$)

Type of care	Perspective	Types of cost	Amount BDT (US$)	Proportion of total cost (patients)
Inpatient Care (*n* = 402)	Provider	Direct medical	88.27 (1.12)	1.02
		Direct non-medical	2015.82 (25.68)	23.24
	Household	Out of pocket payment	2760 (35.36)	31.82
		Indirect cost	3811 (48.55)	43.93
	Societal	All costs	8675.09 (110.51)	100%
Outpatient Care (*n* = 399)	Provider	Direct medical	28.16 (0.36)	1.52
		Direct non-medical	57.8 (0.74)	3.12
	Household	Out of pocket payment	609 (7.76)	32.85
		Indirect cost	1159 (14.76)	62.51
	Societal	All costs	1853.96 (23.62)	100%
All- patient Care (*N* = 801)	Provider	Direct medical	58.21 (0.74)	1.1
		Direct non-medical	1036.81 (13.21)	19.66
	Household	Out of pocket payment	1689 (21.51)	32.02
		Indirect cost	2490 (31.72)	47.21
	Societal	All costs	5274.02 (67.18)	100%

### Annual economic burden

In the light of the earlier findings this section expresses the overall economic burden of diarrhea in Bangladesh. According to the latest national health bulletin, approximately 2.56 million diarrheal cases and 24 deaths were reported in 2015 in various health facilities in Bangladesh [47]. During the hospital based survey, approximately 44% of the diarrheal patients received inpatient hospital care, and 66% had outpatient services. The total annual cost of treatment was US$ 172.02 million for societal perspective while US$ 35.72 million was incurred by the health facilities.

## Discussion

Diarrheal disease is a major public health concern associated with significant morbidity and mortality and economic loss in many societies. While the cost of illness for other infectious diseases in Bangladesh has been investigated [38, 48], knowledge of the cost of illness of diarrheal disease considering the broader societal perspective is limited. The current standards for cost-effectiveness analysis recommend to use a broader societal perspective considering both the provider and household perspective [33].

We found the average length of the diarrheal episode is 5 days (results not presented here) which incurred an average cost of BDT 5274.02 (US $ 67.18) that could be saved if the diarrheal disease was prevented. More than 52% of the total costs are the direct costs borne by the households (31%) and hospitals (21%) as the public hospitals are highly subsidised in Bangladesh [49]. In early 2001, Ali et al. found that the provider cost per day for the management of inpatient and outpatient in a district hospital (Manikgonj) near Dhaka city was BDT 317.27 or US $ 4.04 and BDT 53.74 or US $ 0.69 respectively [28]. Das et el estimated that the average inflation adjusted diarrheal treatment cost for under 5 year old children in rural Bangladesh was US $ 6.99 though they did not consider the laboratory cost borne by the hospitals as well as the income loss of the household [29]. From a multi-country analysis, Rheingans et al. found that the average household treatment cost for childhood diarrhoa was US $ 1.82 where direct cost and indirect costs constituted US $ 1.19 and US $ 0.63 respectively in Bangladesh. The limitations of this study was the relatively small sample size and therefore was not representative of the country and that the study was conducted in a surveillance area [30]. An urban slum based study carried out in Bangladesh where the incidence of diarrhea is high and found that the cost of childhood diarrhea per episode ranged from BDT 124 (US $ 1.81) to BDT 276 (US$ 4.00) with an average duration of 3.76 days of diarrhea [31]. However, all of these studies did not consider the societal perspective and our study expresses a more complete accounting of all the relevant costs associated with an episode of diarrhea.

The current study found that the societal cost of illness per episode was US $ 110.51 for inpatients and US $ 23.6 for outpatients respectively. Recently, similar findings have been observed in a number of LMI countries. Aikins et al. in northern Ghana found that from the health sector perspectives, the average inpatient and outpatient treatment costs were US $ 97.40 and the US $ 4.10 respectively [50]. In Rwanda, the treatment cost per diarrheal hospitalization was US $101 and 65% of this cost was borne by households [51]. Another study conducted in several hospitals in Vietnam found that the average treatment cost per episode was US$ 106.9 whereas indirect costs made up the largest share (51.3%) followed by the direct medical costs (33.8%) and direct non-medical costs (14.9%) [52]. The current study estimated the possible costs of providers (both medical and non-medical) and costs borne by the patients and their caregivers (both direct and productivity loss) in a standard hospital-based survey of six district hospitals of each six divisions in Bangladesh.

The study showed that the treatment cost for outpatients is lower than for inpatients. Although this study did not capture the cause of treatment-seeking behaviour, systemic literature review on the etiology of diarrhea, Walker et al. found ETEC and *V cholerae* O1/O139 to be the most frequently isolated pathogens in inpatients, whereas in the outpatient setting, salmonella sp., shigella sp., and *E histolytica* were commonly found [53]. Several studies conducted in Bangladesh to isolate pathogens from diarrheal stools found shigella sp. in outpatients. However, different types of pathogens such as salmonella sp., shigella sp., ETEC and *V cholerae* O1/O139, rotavirus, giardia, *E histolytica, V parahaemolytica* and campylobacter were typically found in the inpatient setting [54–56]. Further studies are therefore required to better understand these findings.

Among all patients, adults with diarrhea consumed significantly more resources than the young which is consistent with earlier findings that high healthcare expenditure is associated with increase in age [57]. Diarrheal cost burden was significantly higher for the poorest than richest households. The main treatment coping mechanisms was the income of the households which was the only source of household’s income. However, the highest cost burden (21.45%) was observed for poorest quintile than richest (4.21%). Considering the provider cost of treatment, the main cost driver was staff salaries (operating expenditure) and the cost of capital including building cost (investment cost). Some of those investment costs occurred at the beginning of the program and are often not listed in accounts or budget of the hospitals but nevertheless we consider that they are real costs and should be accounted for [35].

We estimate the annual economic burden of diarrheal diseases to be US $ 172.02 million which was 12.28% of the total health expenditure in Bangladesh [58]. However, the estimation is based on the reported cases from health facilities although it is very common that diarrhea is inadequately managed at household level and is associated with high morbidity and mortality. In that sense, we underestimated the actual burden of diarrheal disease in Bangladesh. However, the study was unable to compare the total annual economic burden of diarrheal disease with other settings, however, a literature review of economic burden of rotavirus disease study in Asian settings showed that the annual economic burden of rotavirus illness laid between US$ 0.41 million (Uzbekistan) up to US $ 365 million in China [59], while the annual economic burden of rotavirus exceeds US $ 72 million in India [60]. An unpublished estimation showed that approximately US$ 7.06 million could be saved by preventing rotavirus diseases in Bangladesh [61]. The latest estimate of the annual GDP per capita in Bangladesh (2016) was US 1466 which indicated that approximately 4.58% of GDP per capita spent on treating each diarrheal episode which might be a critical concern as it is the prime cause of hospital admission in Bangladesh [17, 62]. Therefore, by controlling diarrheal diseases huge amounts of resources would be saved. Consequently with reduced number of patients, hospitals could save extra resources like hospital bed, space, doctor’s time, and other resources that could be channeled for other purposes. During the treatment, reliance on OOP expenditures leads to catastrophic economic burden for many households. Further, many poor and vulnerable people cannot afford healthcare as currently there are no social health protection schemes in Bangladesh. To reduce financial barriers to healthcare for the needy and to avoid catastrophic health expenditures, social health protection might be an option which is the core theme of universal health coverage.

The limitations to this study include the design; as a cross-sectional study, it was not possible to estimate the cost variation in light of seasonality such as the incidence of usual peaks during the hot and winter seasons in Bangladesh [63]. The treatment of diarrheal disease relies heavily on households’ treatment patterns and resources which are not covered in this study [50]. The current study was conducted among hospitalized patients, but many diarrheal episodes occurred in the community which is not captured in this study. The other limitation was the sample size as only selected hospitals were considered, albeit on a randomised basis, and therefore the study might not be representative of the whole country. We did not collect the information about severity of diarrheal illness directly, though patients with severe disease are more likely to be inpatients that outpatients [64].

## Conclusions

In LMI countries like Bangladesh, diarrheal diseases continue to be an overwhelming problem. Cost analysis of diarrheal diseases is required for estimating resources for managing and preventing diarrheal disease. Therefore, the economic impact of any public health interventions (either preventive or promotive) that can reduce the prevalence of diarrheal diseases can be estimated from the data generated from this study.

## Abbreviations

ANOVA: Analysis of Variance
BDT: Bangladeshi Taka
HH: Households
HRD: Hospital Records Departments
LMI: Lower and Middle Income
NGOs: Non-Governmental Organisations
OOP: Out-of-Pocket
OOPP: Out-of-Pocket Payment
US$: United State Doller
WHO: World Health Organization
